# Draft Genome Sequence of Magnesium-Dissolving *Lactococcus garvieae* A1, Isolated from Soil

**DOI:** 10.1128/genomeA.00386-17

**Published:** 2017-05-25

**Authors:** Gonca Altın, Emrah Nikerel, Fikrettin Şahin

**Affiliations:** Yeditepe University, Department of Genetics and Bioengineering, Istanbul, Turkey

## Abstract

The probiotic bacterium *Lactococcus garvieae* A1, isolated from soil, is interesting for biomining applications. Here, we report the draft genome sequence and annotation of this strain, with a focus on metal transporter enzymes.

## GENOME ANNOUNCEMENT

Lactococci are Gram-positive, catalase-negative, nonmotile cocci found singly, in pairs, or in chains, belonging to the group of lactic acid bacteria. *Lactococcus* spp. are the usual workhorses of the dairy industry and are used in single-strain starter or mixed-strain cultures (1–3). They are commonly consumed as probiotics, due to their beneficial features for health and nonpathogenic nature. Biomining aims to extract desired minerals or eliminate impurities from ores by using different microorganisms instead of traditional methods of extreme heat or toxic chemicals, having therefore a much lower negative impact on the environment (4, 5). Despite the fact that lactic acid bacteria would be promising organisms for biomining, simply due to their medium acidification properties, they have seldom been used in biomining studies. Recently, we reported on the *Lactococcus garvieae* A1 strain for its use in removing calcium carbonate impurities in magnesite. Interestingly, next to the above-listed uses for, e.g., probiotics, this strain exhibits strong Ca-dissolving capacity well beyond its potential due to medium acidification alone (6). To understand the biomining mechanism of *Lactococcus garviae* strain A1, we carried out a whole-genome sequencing and annotation study of this bacterium isolated from the soil. Here, we report the genome sequence of this strain.

Genomic DNA of strain A1 was isolated using a Genomic DNA isolation kit (Invitrogen), according to the manufacturer’s protocol. The sequencing was performed using an Illumina HiSeq 2000 sequencing platform (Illumina, Hayward, CA, USA) at Macrogen, Inc. A total of 18,275,986 paired-end reads were obtained, each 100 bp long and 250 bp apart. Quality-filtered clean reads were assembled by the CLC Genomics Workbench assembly pipeline. The reads were assembled into 25 contigs, with a median coverage of 901-fold, which resulted in a genome size of 2.04 Mb with a G+C content of 38.0% (contig length threshold, 200 bp; contig coverage threshold, 100×). The longest contig is 514.1 kb, and the *N*_50_ and *L*_50_ were found to be 207.8 kb and 4, respectively. The ﬁnal assembly was automatically annotated using the RAST annotation pipeline. The genome of the A1 strain contains 2,007 predicted coding sequences (CDSs). Genome analysis revealed that *L. garvieae* A1 contained several genes encoding an ABC transporter for metal ions; lead-, cadmium-, zinc-, and mercury-transporting ATPases (EC 3.6.3.x); and cation-transporting ATPases, which can all be attributed to the biomining activities, in addition to the acidic fermentation metabolism. In line with the isolation media, the organism also has genes encoding several enzymes, including those for lipase, protease, and glucosidase activity.

Here, we report the ﬁrst complete genome sequence for the *L. garviae* A1 strain. Further *L. garviae* transcriptome analysis and comparisons will elucidate the factors involved in biomining and the complete magnesite biomining mechanism of this strain.

### Accession number(s).

This whole-genome shotgun project has been deposited at DDBJ/ENA/GenBank under the accession no. NBBK00000000. The version described in this paper is version NBBK01000000.
